# An Intervention Based on Acceptance and Commitment Therapy for Childhood Separation Anxiety: A Case Study

**DOI:** 10.3390/bs16010082

**Published:** 2026-01-07

**Authors:** David Lobato, Juan Miguel Flujas-Contreras, Francisco Montesinos, María M. Montoya-Rodríguez

**Affiliations:** 1 Departamento de Psicología, Universidad Europea de Madrid, Calle Tajo S/N, Villaviciosa de Odón, 28670 Madrid, Spain; francisco.montesinos@universidadeuropea.es; 2 Departamento de Psicología, Universidad de Almería, 04120 La Cañada, Spain; jm.flujas@ual.es; 3 Departamento de Psicología, Universidad Católica del Uruguay, Montevideo 11600, Uruguay; maria.montoya@ucu.edu.uy

**Keywords:** anxiety, family, psychological flexibility, acceptance, child, parents, ACT

## Abstract

This study illustrates the application of Acceptance and Commitment Therapy (ACT) for a 12-year-old boy with separation anxiety and his mother. Over 23 sessions, ACT strategies promoted psychological flexibility, values-based parenting, and adaptive behaviors. The intervention reduced the child’s experiential avoidance, anxiety, and depressive symptoms, while increasing value-oriented actions, while the mother showed improved psychological flexibility and life satisfaction. The results were sustained at a three-month follow-up. This case study highlights the potential of ACT in treating childhood separation anxiety by simultaneously involving parents, demonstrating its feasibility and efficacy. The findings provide guidance for adapting ACT for families and child populations.

## 1. Introduction

Fear and worry are normal aspects of childhood development. However, for some children, these problems aggravate and interfere with their adjustment to different contexts in life (Breinholst et al., 2012). Anxiety disorders affect 10–20% of children and adolescents globally, often co-occurring with depression and impacting social, academic, and family functioning (Costello et al., 2005; Cummings et al., 2014; Rapee et al., 2023). Notably, recent epidemiological trends indicate increasing incidence rates of anxiety disorders in children and adolescents, particularly in the post-COVID-19 era, with some studies reporting prevalence increases of 15–25% compared to pre-pandemic levels (Bie et al., 2024; Racine et al., 2021). Additionally, anxiety disorders in children and adolescents are associated with a risk of having recurrent episodes during adulthood (McLaughlin & King, 2015), along with other behavioral problems such as substance addiction or major depression (Kushner et al., 2012). Separation anxiety is one of the most common and impairing anxiety presentations in childhood (Feriante et al., 2023), forming the specific focus of the present case study. Parenting practices strongly influence the development and persistence of childhood anxiety (Sanders et al., 2021). Family members usually respond to a child’s needs, attempting to reduce their suffering and make their integration into different contexts easier. Such family accommodation can help the child with integration (Lebowitz et al., 2013). However, if the parents’ response becomes excessive, rigid, or severe, it may have a direct connection to the child’s development of anxiety (Iniesta-Sepulveda et al., 2021).

Standard evidence-based treatments for childhood anxiety typically include cognitive–behavioral therapy (CBT), which combines procedures such as graded exposure, cognitive restructuring, skills training, and behavior-modification strategies. Although effective, these approaches can show variable long-term outcomes and may not fully address contextual maintenance factors, such as parental accommodation or experiential avoidance (Perkins et al., 2023). In contrast, Acceptance and Commitment Therapy (ACT; S. Hayes et al., 2014) has demonstrated efficacy across diverse psychological conditions. Meta-analytic evidence shows that ACT is effective not only for anxiety and depression but also for chronic pain, substance use disorders, obsessive–compulsive disorder, and post-traumatic stress disorder, with effect sizes comparable to or exceeding those of CBT in some conditions (A-Tjak et al., 2015; Gloster et al., 2020).

Acceptance and Commitment Therapy (ACT; S. Hayes et al., 2014) addresses family accommodation and parental regulation of discomfort to support child development. The basis for ACT is the Relational Frame Theory (RFT: S. C. Hayes et al., 2001). In the parenting field, RFT focuses on the analysis of verbal behavior and its dysfunctional regulation of parental upbringing practices, including verbal family rules, experiential avoidance, and value-oriented behavior. ACT has shown promising results in addressing psychological and medical issues, as well as in parental programs by promoting parents’ psychological flexibility in managing children’s anxiety problems (L. Coyne & Murrell, 2009; McCurry, 2015; Swain et al., 2015; Perkins et al., 2023; Whittingham & Coyne, 2019).

Children naturally encounter challenges that can distress their parents, often leading to controlling or avoidant behaviors (Flujas-Contreras et al., 2022). When psychological challenges arise, parents may overidentify with distressing thoughts, emotions, or memories, treating them as facts (S. C. Hayes & Greco, 2008). Because of this, the way in which parents relate to their own verbal regulations may influence their parenting practices, maintaining or aggravating their child’s behavior problems (L. W. Coyne et al., 2011); in addition, their practices can reduce their child’s compliance with pro-therapeutic instructions, often by assuming a psychological flexibility (PF) in their parents, and therefore undermining the effectiveness of the intervention (Raftery-Helmer et al., 2016).

Parental PF is defined as the ability to deal with unpleasant private events (stress, change processes in children, parenting difficulties, or fear) without implementing controlling or suppressive behaviors, and guiding behavioral repertoires toward valuable parenting in their interactions with their children (Brassell et al., 2016; Fonseca et al., 2020). On the contrary, psychological inflexibility (PI) in parents leads to avoidance and controlling behaviors, disrupting the relationships they aim to build with their children. This type of response is effective in the short term, but the psychological discomfort increases in the long term (S. Hayes et al., 2014), which can cause parental stress and anxiety (Daks et al., 2020; Fonseca et al., 2020), depressive episodes (Biglan et al., 2015), and/or inconsistent or maladaptive habits (Sairanen et al., 2018). Parental PF is related to fewer behavioral and emotional problems in children and family adjustment (Burgdorf & Szabó, 2021; Emerson et al., 2021; Whittingham & Coyne, 2019), and family PI is characterized by the creation of difficulties for children in exercising psychological acceptance (Williams et al., 2012), anxiety (Ciarrochi et al., 2010), adjustment to contexts (Moyer & Sandoz, 2015), hyper-controlling tendencies, avoidance of stressful situations, and increased family maladjustment (Fonseca et al., 2020).

ACT is particularly useful in child and adolescent therapy because of the characteristics of the intervention, which are typical of the functional contextualism paradigm. For example, the use of metaphors allows contact with an avoided stimuli, supporting the involvement of the child and decreasing experiential avoidance strategies and functional equivalence relations. Because of its strategies, it also supports the formation of a self-as-context approach that enables other PF processes to take place. Another example can be seen in experiential intervention, which reduces the language trap and facilitates the child’s involvement in the therapeutic process; otherwise, a verbal exchange can be conditioned and can produce an aversive response. In other words, experiential, evocative, and playful exchanges often facilitate the acquisition of new skills and habits (O’Driscoll et al., 2020).

All of the above highlights the importance of promoting PF as a useful strategy to help parents facilitate family-accommodation processes, reduce controlling behaviors, respond in a way that is focused on environmental contingencies and on their child’s needs, and guide parents’ behavioral repertoires towards values. The aim of this article is to present a case that illustrates how ACT can be applied to clinical methods to promote PF in a mother and child simultaneously, so that ACT’s viability and usefulness and the specific contribution of this method can be shown. Case studies are particularly well suited for examining clinical processes in depth and for illustrating how interventions such as ACT can be implemented in real-world therapeutic contexts. This case study is presented with the intention of providing a model for clinicians who wish to adapt ACT for families and for the child and adolescent population.

## 2. Case Description

### 2.1. Treatment, Setting, and Context

The treatment took place at a private psychological clinic after the mother, following her primary care physician’s recommendation, sought help for her son’s anxiety and fear issues. The middle-class family, living in a residential area on the outskirts of the city, consisted of a 40-year-old mother, a 45-year-old father, a 16-year-old son, and a 12-year-old son (here, the latter is referred to by the pseudonym Miguel; this is the child for whom the psychological intervention was requested). The intervention was delivered by a licensed therapist who specializes in functional contextualism (the first author of the manuscript), with clinical experience delivering ACT-based interventions for children and families.

Written informed consent was obtained from the participants. The study gained the endorsement of the Ethics Committee of the Universidad Europea de Madrid; approval number, CIPI/20/153. All identifying information was removed or modified to prevent traceability; pseudonyms are used for both mother and child, and all data have been stored securely with access restricted to the research team.

### 2.2. Patients: Child and Mother

The family was of Spanish origin, and Spanish was their primary language. Miguel had not received prior psychological treatment beyond the referral from the Child Mental Health Services. Miguel, with a history of heart disease and extended hospital stays, has long shown anxiety and difficulty bonding with peers, along with a persistent fear of sleeping alone. His mother reported academic difficulties, including repeating a grade due to medical issues. A year ago, his anxiety worsened after a stressful episode at camp. This fear extended to the school setting, where frequent truancy occurred as his mother often picked him up early. His mother reported a significant reduction in social involvement in activities with peers and a progressive withdrawal from hobbies such as martial arts and soccer. Currently, he reports somatic symptoms, such as stomachaches and headaches, along with a fear that something bad might happen to his mother or that she might die. He spends most afternoons with his mother, watching series and playing board games or video games. He attends therapy with a motivated outlook, expressing a desire to ‘get rid of fears’ and feel happy again. He had been referred to the clinic with a formal diagnosis of separation anxiety from Child Mental Health Services, and a full psychological assessment was conducted at intake to characterize his functioning and to establish a baseline for evaluating therapeutic change. This diagnosis had been established by a psychiatrist through a non-structured clinical interview based on DSM-5 diagnostic criteria

The mother had been unemployed since Miguel developed heart problems. She has structured her life around caring for him, focusing on his happiness. She reported that his fear-driven behaviors, complaints, and growing isolation cause her significant anxiety and feelings of helplessness. To reduce his discomfort, she frequently picks him up early from school, spends afternoons with him, or distracts him with activities. The youngest of four sisters, she grew up under her own mother’s strict supervision and control. Currently, she has minimal social contact or leisure activities. Although no formal clinical diagnosis was established for the mother, her psychological functioning and progress were systematically assessed at intake and throughout the intervention.

### 2.3. Family Interactions

The father said that he did not want to participate in the therapeutic process, blaming his son’s fears on his mother’s responses, which he described as irrational or excessive. The children do not spend time together, neither do the parents, and the father does not spend time with the son who goes to therapy. He displays punitive and hostile behavioral repertoires in front of the child whenever he shows anxious behaviors, such as recrimination or blaming the mother. The mother displays behavioral repertoires of protection and care such as staying with him at home doing his homework, picking him up from school when the school calls her, staying at home instead of going out with her friends, or going with him when he calls her.

According to the functional analysis of behavior, the mother–child relationship is characterized by the absence of activities with appetitive function in the mother’s context, the formation of the maternal identity based on caregiving behaviors, the dysfunctional relationship with her partner, the history of early hospitalizations, and the family’s learning of controlling behaviors. These characteristics increase the probability of accommodative behaviors being displayed as a response to the child’s anxiety, relieving it in the short term but reinforcing it in the long term and causing a loss of opportunities for the child.

## 3. Intervention Procedure

### 3.1. Overview of Treatment

The treatment included a joint pre-intervention assessment session, 10 sessions with the mother, 10 sessions with Miguel, a joint postintervention session, and a follow-up session three months later. Each session lasted one hour and was conducted weekly, totaling 23 sessions. Treatment followed a structured ACT-based protocol that included values clarification, cognitive defusion, acceptance-based strategies, and committed-action tasks adapted to the separation-anxiety-related triggers that were identified in the functional assessment. Illustrative examples of ACT processes were incorporated throughout the intervention. For instance, when Miguel said “my fear controls me”, the therapist guided him to observe this as a thought rather than a defining truth, facilitating a shift from self-as-content to self-as-context. During the values work, Miguel identified “being independent”, which supported small committed actions such as remaining at school a few extra minutes. With the mother, statements such as “I must be with him so he doesn’t suffer” were used to introduce defusion and to reorganize contingencies away from avoidance and toward value-consistent behavior.

The number of sessions was not predetermined but adjusted to Miguel’s clinical progress and the functional needs that were identified throughout treatment, consistent with ACT’s flexible and process-oriented approach. Daily behavioral self-monitoring activities were reviewed; they were received by email and used to refine the functional analysis with both mother and child during the sessions, to train behavioral self-discrimination skills, and to identify changes in approach and avoidance behaviors linked to the identified values. Daily behavioral self-monitoring records were used as idiographic clinical monitoring tools within a functional-analytic framework, rather than as qualitative data for formal or reproducible qualitative analysis. The mother and child were treated separately to target distinct behavioral processes—parental accommodation, experiential avoidance, and Miguel’s anxiety-related avoidance—and to provide space to address the mother’s own difficulties and guide her in managing contingencies around Miguel’s approach and avoidance behaviors. This structure allowed each to develop the necessary skills individually and reduced the risk of reinforcing avoidance within the sessions.

The following subsections provide a detailed breakdown of the sessions and the psychotherapeutic procedures used.

### 3.2. Initial Assessment

Session 1 involved a joint pre-treatment evaluation conducted through a psychological interview and psychometric tests for both the child and the mother. The parents also signed consent forms for participation and the potential use of the data for research purposes. Here, we describe the tests that were administered during this assessment.

#### 3.2.1. Measures Used to Assess the Child

Avoidance and Fusion Questionnaire for Youth (AFQ-Y; Greco et al., 2008): The Spanish version (validated by Valdivia-Salas et al. (2017)) of the instrument was used to assess cognitive fusion and experiential avoidance. This instrument consists of 17 items on a 5-point Likert scale that assesses the tendency to suppress, control, and escape from unpleasant private events (experiential avoidance subscale) and the tendency to become entangled with private verbal control (cognitive fusion subscale). This assessment derived a Cronbach’s alpha of 0.87 for the total score, and 0.81 and 0.76 for the respective subscales. The higher the score, the greater the tendency to experience experiential avoidance and cognitive fusion.

Willingness and Action Measure for Children and Adolescents (WAM-C/A; Larson, 2008): The Spanish version (validated by Cobos-Sánchez et al. (2020)) was used. This method assesses a child’s willingness to experience unpleasant private events without displaying avoidance or escape behaviors (willingness) and value-oriented behavioral repertoires (action). It consists of 14 items on a 5-point Likert scale. This assessment derived a Cronbach’s alpha of 0.78 for the total score, a Cronbach’s alpha of 0.81 for the willingness scale, and 0.71 for the action scale. The higher the score, the greater the tendency to be willing to engage in private events and in value-oriented behaviors.

Children’s Depression Inventory—Short (CDI-S; Kovacs, 1992): This evaluates the symptomatology and intensity of negative affects. The Spanish version (made by Del Barrio et al. (2002)) was used. It consists of a Likert-type scale that is scored from 0 to 2 and has a Cronbach’s alpha of 0.71. A higher score indicates a higher negative affect.

Spence Child Anxiety Scale (SCAS; Spence, 1997): The Spanish version (validated by Orgilés et al. (2012)) was used. It is made up of 38 items that assess anxiety symptoms using a 4-point Likert-type scale that is scored from 0 to 3. It also includes six positive filler items to counteract the negative bias of the previous elements. These filler items are not scored or considered in the analyses. This scale assesses the six most common anxiety disorders in childhood, and a higher score indicates a higher level of anxiety. The psychometric properties of the scale are good with Spaniards, with high internal consistency (Cronbach’s alpha = 0.89).

#### 3.2.2. Measures Used to Assess Mother

Parental Acceptance Questionnaire (6-PAQ; Greene et al., 2015): The Spanish version (validated by Flujas-Contreras et al. (2020)) was used. This questionnaire evaluates parental PF. The Spanish version of the questionnaire consists of 16 items on a 4-point Likert scale. The factors explored suggest that this questionnaire has a three-factor structure (Gootzeit, 2014; R. Harris, 2019). This assessment derived a Cronbach’s alpha of 0.81 for the total score. The factors are open response style, aware response style, and active response style. The subscales have an acceptable internal consistency, with Cronbach’s alpha ranging from 0.66 to 0.71. The higher the score, the higher the PI.

Satisfaction with Life Scale (SWLS; Diener et al., 1985): The version validated in Spanish by Vázquez et al. (2013) was used. It consists of five items, evaluated with a scale from 1 to 7, that assess life satisfaction. The scale in the Spanish version has a Cronbach’s Alpha of 0.82. The higher the score, the higher the life satisfaction.

### 3.3. Intervention

The intervention spanned from session 2 to session 10 and followed a similar structure for both the mother and the child. Sessions 2–4 focused on identifying and understanding functional behavioral classes that sustain psychological problems. Sessions 5–8 emphasized the self-discrimination of private events, framed within the deictic “I”. Finally, sessions 9 and 10 centered on fostering flexible, value-oriented behavioral repertoires. Here, we provide a detailed description of the mother–child intervention approach, the PF processes that were worked on, and the therapeutic resources used. Although the intervention was designed to be flexibly paced, the planned objectives for each stage were completed within the session ranges described above. Progression across stages remained functionally guided and was adjusted weekly based on behavioral self-monitoring records, observed avoidance patterns, and the participants’ performance during experiential and values-based exercises. In addition, procedures were tailored to the child’s idiosyncratic stimulus universe to enhance engagement and functional relevance—for example, incorporating his preferred comic-book characters during values clarification (e.g., Batman) and using familiar fictional figures (e.g., from Star Wars) during mindfulness and self-as-context training exercises. This individualized and functionally informed approach ensured the fidelity to ACT processes while maximizing practical applicability.

The intervention with the mother is described first, followed by that with the child.

#### 3.3.1. Intervention with the Mother: Sessions 2–4

These sessions analyzed the functional relationships between the mother’s behavioral repertoires and their short- and long-term effectiveness in managing her child’s anxious behavior. This included examining the mother’s verbal rules within a historical and contextual framework. Metaphors were used to highlight values that could guide her parenting process, along with exercises in creative hopelessness. Here, we provide a summary of the therapeutic tools employed.

Analysis of interactions: A behavioral self-monitoring record of mother–child interactions with their different contexts was filled in daily; after several weeks, a functional analysis of the anxious behaviors was performed. In these self-monitoring reports, the mother wrote down difficult situations that occurred on each day, unpleasant private events, and the discriminant function these had on experiential avoidance behavior, as well as their effect on her child’s behavior. Behavioral self-monitoring was also included for school-related events. These records allowed us to carry out a functional analysis of the child’s anxiety, with the aim of making the mother aware (self-discriminating) that her private events had an experiential avoidance function, as they translated into controlling or suppression behavior of her own and her child’s discomfort, impacting on the anxiety problems and creating a loss of vital opportunities for her child. A brief ABC-structured summary of one example or event, derived based on the behavioral self-monitoring records, is presented here: (A) when Miguel showed distress (e.g., Miguel tells his mother that he is not feeling well, and she notices that her son becomes nervous) and the mother experienced aversive private events such as fear, guilt, or the rule-governed belief that “a mother must not allow her child to suffer”; (B) she engaged in accommodation behaviors such as picking him up early from school, staying home with him, or reorganizing routines to reduce his discomfort; (C) these responses produced immediate relief from her own aversive private events and, in many interactions, increased her sense of closeness and affection from Miguel. These are functions that negatively and positively reinforced the behaviors in the short term, while contributing in the long term to reduced opportunities for Miguel to engage in autonomous learning, increased family distress, strengthened anxiety-related behavioral classes, and decreased Miguel’s participation in broader school and social contexts

Values clarification: These strategies, from functional contextual science practices, allow parents to be guided by valuable actions rather than by avoidance or escape from unpleasant events (L. W. Coyne et al., 2011). Thus, they can be implemented to reduce one’s control over unpleasant private events and, when necessary, supports people in displaying behaviors that guide valuable parenting (Blackledge & Drake, 2013), even if this means experiencing anxiety as a mother. For the clarification of values on which to guide parenting, two exercises were conducted: “What kind of mother do you want to be?” (L. Coyne & Murrell, 2009); “Prime-Time News Story” (Stoddard & Afari, 2014), rephrased in support of the type of parenting that we were seeking to facilitate in this approach. Based on the findings gathered in the sessions, the mother stated: “I see him sharing his life with whomever he wants and who makes him happy, not choosing a girlfriend because he’s scared of being alone. I see him traveling, studying abroad, discovering the world. I see him with friends and with lots of plans every day”. Therefore, the values of promoting autonomy, sociability, and the ability to make choices were identified.

VOI-VOI Triangle (VOI stands for “assess, observe, investigate” in Spanish; Mandil, 2022): Once the mother’s values were identified, the private events that cause her to engage in experiential avoidance behaviors and the results of these on her child’s anxiety were linked through the VOI-VOI triangle. This makes it easier to connect with one’s own painful experiences, which relate to the reason for going to therapy, the practice of perspective-taking, and the ability to encourage effective parental repertoires for flexible child development (L. Coyne & Murrell, 2009).

Creative hopelessness: The therapist and the mother explored the nature of the mother’s behavioral struggle with anxiety. Using the previously described strategies, the behaviors and their effects on reducing her child’s anxiety problem were exemplified. The mother was then asked to evaluate the short- and long-term effectiveness of the repertoire employed, as well as their possible associated impacts. Having explained the values, each time the mother described a behavior related to her child’s anxiety behavioral repertoire, an attempt was made to help her to define the behaviors that reinforce Miguel’s problem, assessing their usefulness, and determining whether they point towards or distance from the related value.

#### 3.3.2. Intervention with the Mother: Sessions 5–8

In these sessions, the mother was encouraged to respond in a more flexible way to her private events, expanding her behavioral repertoire and orienting them towards valuable motherhood, even if this meant being uncomfortable. To this end, training was performed in the following areas:

Shaping the verbal labeling of private events: This training aimed to enhance the mother’s self-discrimination and improve her psychological flexibility (Whittingham & Coyne, 2019). Each time the mother described a public behavior, she was guided to identify the private event with a discriminant function, such as fear, distress, or thoughts of being a bad mother. These private events were also brought into the therapeutic context to support her in practicing her self-discrimination skills.

Transitioning from self-as-content to self-as-context: This training involved creating psychological distance from private events such as unpleasant thoughts or emotions, which often serve as triggers for controlling behaviors. When such repertoires negatively affect mother–child interactions, they become inflexible and hinder a child’s development, moving away from valuable parenting practices (L. W. Coyne & Wilson, 2004). To reduce this verbal control of private events, the mother was trained in psychological acceptance and defusion through the following approaches: (a) the swamp metaphor (Whittingham & Coyne, 2019), to promote a context of acceptance; (b) defusion training, which included steps such as noticing physical changes in the body, identifying and naming emotions, practicing conscious breathing, allowing herself to feel emotions without judgment, and reflecting on how to act in a way which would be coherent with her parenting values.

#### 3.3.3. Intervention with the Mother: Sessions 9 and 10

During these sessions, the focus was on practicing more flexible alternative repertoires, emphasizing values-guided behaviors. The aim was to ensure that behavioral changes aligned with the mother’s values, allowing the transformation of stimulus functions and maintaining these functions even in the face of aversive contingencies. This process involved the following measures:

Identifying committed actions: The therapist helped the mother in identifying actions that are consistent with her values of wanting autonomy, sociability, and decision-making abilities for her child.

Taking small, daily steps: The mother was encouraged to engage in daily behaviors that embodied her values, even when faced with unpleasant private events. This approach was taken with the aim of reducing the influence of private events on public behaviors and to transform the aversive nature of those behaviors.

#### 3.3.4. Intervention with the Child: Sessions 2–4

The main objective of these intervention sessions was to activate value-oriented behavior in the presence of unpleasant private events (anxiety). For this purpose, and similarly to the approach that was taken with the mother, we worked on identifying the functional relationships between the behavioral repertoires of control or avoidance of anxiety and the contingencies that maintained them; moreover, we sought to connect them with their ineffectiveness in the long-term elimination of suffering. Value clarification exercises were adapted to the child’s context and creative hopelessness was induced. The therapeutic processes employed are described below.

Analysis of interactions: A behavioral self-monitoring approach designed for children and adolescents, known as the chain breaker (Mandil, 2022), was used. Efforts were made to encourage the child to engage in self-monitoring surrounding his operating problems, supported by the following questions: (a) “What was happening in that moment? What were your mind and body telling you?”—these questions were used to identify stimuli, whether public or private, with an experiential avoidance function; (b) “How did you solve the problem?”—with this question, we sought to identify the behaviors that the child displays when trying to solve a problem; (c) “What happened next?”—this allowed us to analyze the consequences and personal impacts of the behavioral repertoire. A brief ABC-structured summary, derived based on Miguel’s behavioral self-monitoring records, is presented here: (A) separation cues, somatic discomfort such as stomachaches, or catastrophic thoughts such as “something bad is going to happen to my mother” occur; (B) then, he engaged in avoidance or escape behaviors, including staying physically close to his mother, refusing to attend school, or withdrawing from social activities; (C) these responses produced immediate reductions in anxiety that negatively reinforced the avoidance pattern in the short term, while contributing to increased dependency, maintenance of anxiety-related behavioral classes, reduced opportunities for valued and autonomous action, and diminished participation in school and peer contexts in the long term.

Values clarification: To explain the values, we first tried to link the “choose what matters, do what matters” process with the reason for going to therapy (Black, 2022). That is, the anxiety-regulating behaviors exhibited by the child were identified as increasing his discomfort and making him feel worse, causing the problems to occur in other contexts and distancing him from the things that matter to him. Subsequently, Miguel was asked to identify a comic book character that he admired for what they do and to respond by describing the typical behaviors of that character. This was a way of identifying some actions that might be related to the values that were meaningful to the child. After this exercise, the following values were identified: “I want to be a fun friend that signs up for plans; to be able to travel with them when I’m older and help others”.

VOI-VOI Triangle (VOI stands for “assess, observe, investigate” in Spanish; Mandil, 2022): Once the values and the private events that elicit experiential avoidance behaviors were identified, they were related with values within a triangle: values—“Being fun, traveling and helping people”; what is observed—“I feel very afraid when I have to do something without my mother”; what is investigated—“When I feel this way, I end up staying with her and I don’t make progress on what matters to me”.

Creative hopelessness: Finally, all kinds of behaviors that are functionally equivalent to the avoidance of anxiety were listed and put in a diagram to support Miguel’s understanding of how those actions either helped him to eliminate the problem or escalated it, in addition to determining whether they pointed towards or created distance from his values.

#### 3.3.5. Intervention with the Child: Sessions 5–8

These sessions aimed to equip Miguel with the psychological tools required to respond more flexibly to anxiety-inducing private events, enabling him to act in ways that are consistent with his values rather than being aversive control behaviors. The training included the following measures:

Psychological acceptance through mindfulness: Miguel was encouraged to create space for anxiety, reducing the likelihood of suppression or controlling behaviors. This involved practicing conscious breathing focused on the emotion of anxiety, following three steps: slowing down, adopting a perspective of genuine curiosity, and focusing on the purpose of the anxiety (Cassidy & Coyne, 2022).

Self-as-context training: Miguel practiced his identification of private events using hierarchical and deictic cues through the Wise Warrior exercise (Gómez-Becerra et al., 2019). Key emotions and thoughts identified during the exercise included the following: “Anger with my father”; “Sadness because people do not want to connect with me”; “I am getting bored”; “this is not working”.

Defusion: The goal of this training exercise was to weaken the control of thoughts that triggered avoidance- or anxiety-related behaviors. Miguel externalized his thoughts by writing them down or visualizing them on a screen (R. Harris, 2019). Notable thoughts included the following: “something bad is going to happen to me and there will be no one to help me”; “the fear will be horrible and unbearable”; “I feel fear and stomachache”.

#### 3.3.6. Intervention with the Child: Sessions 9 and 10

The aim was for Miguel to exercise more flexible alternative repertoires to orient his behavior toward his values, despite his anxiety. For this purpose, a clear distinction was drawn between actions, goals, and values. Each day, the child committed himself to a concrete goal-oriented action that correlated with a value. Some significant actions were the following: “Sign up for karate again”; “Chat via WhatsApp with my friends”. Similarly, some goals were set, such as the following: “Regain the friendships I am losing”; “connecting with my values”; “travel with my friends when I grow up” (e.g., Japan).

### 3.4. Postintervention

During the joint postintervention session, progress was evaluated for both participants, with a focus on the extent to which they had developed behavioral repertoires consistent with their values. Three months later, a follow-up session was held to examine the maintenance of these therapeutic outcomes.

## 4. Results

### 4.1. Child’s Results

**Figure 1:**
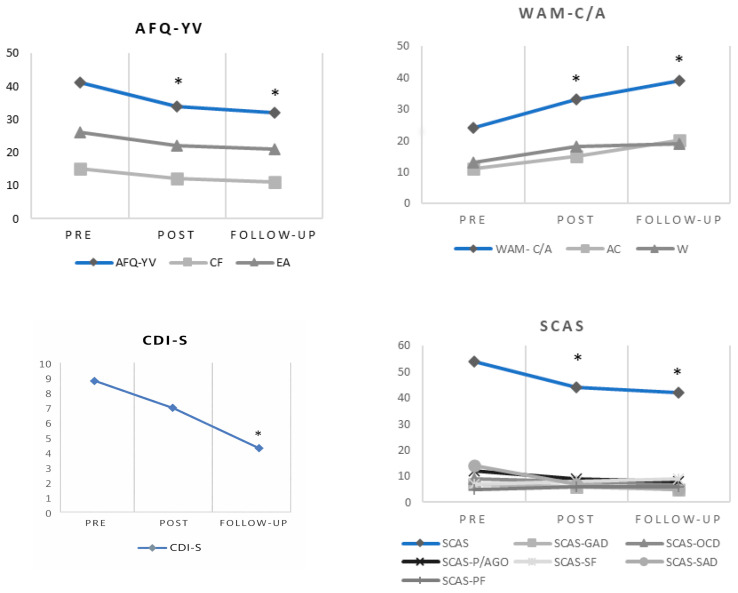
Pretest, post-test, and three-month follow-up scores for the child. Note: * indicates a clinically significant recovery according to criterion “b” of the Jacobson and Truax (1991) method.

Figure 1 shows the direct scores on the instruments before starting the intervention, after the intervention was completed, and during the three-month follow-up. To evaluate the presence of significant clinical changes, the Jacobson and Truax’s (1991) method was used, following criterion b, for which two standard deviations above and below the average of the functional distribution were considered. This rate was calculated for the overall scores of each scale.

Regarding the assessment carried out with self-reports at the initial stage of the intervention with Miguel, the PI score (AFQ-YV) was at a medium range. However, the experiential avoidance (EA) subscale showed elevated scores. Similarly, on the WAM-C/A instrument, scores were below the average on both the total score (WAM-C/A) and the subscale of value-oriented committed actions (CAs). These results support the idea that Miguel exhibited a pattern of experiential avoidance. Some topographies of these kinds of behaviors included staying with his mother, not going out, not meeting with friends, or not going to school whenever he thought that something bad was going to happen to him or his mother. These behaviors acquired an avoidance or escape function from the discomfort.

With respect to the initial measures of depressive symptomatology, the CDI-S scores were at the 40th percentile, indicating that Miguel did not present depressive symptomatology when he started therapy. Anxiety symptoms were measured with the SCAS, finding that the social anxiety and panic/agoraphobia scores exceeded the average score by two standard deviations.

As can be seen in Figure 1, the scores indicate a reduction in the measures of experiential avoidance (EA) and cognitive fusion (CF) after the intervention. The difference between pre- and post-treatment numbers in the overall score of the AFQ-YV scale indicates recovery according to Jacobson and Truax’s method. Moreover, we identified an increase in the willingness (W) and committed action (CA) scores relative to the pretest. The child’s willingness to experience discomfort (WAM-C/A) at the end of the treatment showed a clinically significant recovery. A reduction in depressive symptomatology (CDI-S) was also observed, remaining in the low-percentile range. A clinically significant recovery was observed for global anxiety (SCAS). Most anxiety subscale scores (SCAS) decreased. Post-treatment separation anxiety (SCAS-SAD) and panic attack and agoraphobia (SCAS-P/AGO) decreased to around the normative average.

These results were maintained at the 3-month follow-up. Decreases in experiential avoidance (EA), cognitive fusion (CF), depressive symptomatology (CDI-S), and most of the anxious symptomatology subscales (SCAS) were observed. Moreover, increases in willingness (W) and committed actions (CAs) were observed. These improvements in scores on the AFQ-YV and the WAM-C/A between the pretest and the follow-up represent a clinically significant recovery.

### 4.2. Mother’s Results

**Figure 2:**
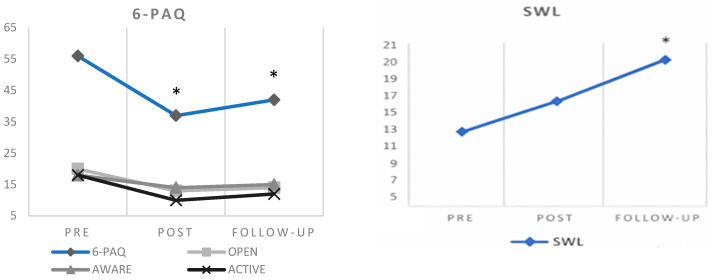
Pretest, post-test, and three-month follow-up scores for the mother. Note: * indicates a clinically significant recovery according to criterion “b” of the Jacobson and Truax (1991) method.

The initial assessment with the mother (Figure 2) reflected a high score in parental PI (6PAQ), with very little openness, awareness, and active coping. These scores indicate that, at times, when the mother had to cope with a discomforting situation with Miguel, her coping style was possibly informed by an avoidance of the discomforting situation or of her child’s suffering. Difficulty was also observed in her ability to be in the present moment when carrying out her parenting practices, and she expressed difficulty in acting in a way which was coherent with her values as a mother. Some examples reported by the mother in the initial assessment were that she stayed with him, picked him up from school or organized her day so that Miguel would always be accompanied when she saw him crying, suffering, or expressing fear. The behaviors in this repertoire generated an implication of experiential avoidance surrounding aspects of her son’s life that she valued, such as his autonomy or sociability.

The SWL scale also reflected low life satisfaction. This score was reflected in the initial interview with the mother; for example, she stated that “I have already thrown my life away, I don’t want my son to live my life, or I want him to experience a more joyful or risky life than mine”.

After the intervention, a reduction in scores was observed on all the subscales of parental PI (6-PAQ), especially in difficulties in maintaining a committed response style (active). On the other hand, an increase in life satisfaction (SWL) was observed compared to the initial assessment, which could be interpreted as “slightly unsatisfied”, although this result did not reach a significant difference. Scores at the 3-month follow-up show a slight increase in PI with respect to the post-test; this increase indicates a clinically significant recovery. Life satisfaction at follow-up shows a further increase to a mid-range score, and this change represents a clinically significant recovery.

In addition to the quantitative findings, brief interviews during the follow-up suggested that qualitative improvements had been made in Miguel’s daily functioning, including a greater degree of autonomy, a gradual re-engagement in social activities, and a general improvement in his mood. Finally, to document qualitative data on the impact of the intervention for Miguel and his mother, during this follow-up phase, they were asked what they had learned from the therapy process. The mother stated the following: “I know that I am very afraid of being a bad mother. I didn’t want to be like my mother was, who let me grow up alone and left me needing to be attached to someone so I wouldn’t feel bad. That worry will always be there, but now I want to promote a different kind of relationship with my son”. Miguel stated the following: “now I am starting to regain the things that made me happy, although I always want my fear to go away, I don’t like it. And it’s still with me, but I’ve learned that I can’t let it take control over me and my life”.

## 5. Discussion

The results of this study point to the usefulness of ACT in working with parents, children, and adolescents to improve family adjustment to their child’s anxious behavior and to promote PF. Miguel showed reduced scores for depressive and anxious symptomatology, as well as for measures of experiential avoidance and cognitive fusion. Additionally, his willingness and committed action scores increased in comparison to those in the pretest. All these improvements were maintained at follow-up. The same trend was observed in the mother, who showed improvements in parental PF and life satisfaction compared to the initial assessment, which were maintained three months after the intervention. These results are consistent with those that have been reported in the literature, where improvements are maintained months after the end of the intervention (Swain et al., 2015). These findings point to ACT being a highly viable treatment for anxiety disorders in both child and adult populations. In this case, the pattern of improvement appears to be driven by alterations in avoidance-based functional classes—through defusion, acceptance, and values-guided action—which reduced the regulatory impact of aversive private events and helped the mother and child to disrupt their avoidance–accommodation cycles; these cycles were identified in the functional analysis, as shown in prior ACT studies examining similar mechanisms of change (Hancock et al., 2016; Settipani et al., 2013; Raftery-Helmer et al., 2016).

There is an increase in the availability of data evidencing ACT’s effectiveness in the context of child and adolescent mental health, medical issues, substance use, neurodiversity, and socially vulnerable populations (Kallesøe et al., 2021; Makki et al., 2018; Pahnke et al., 2014). To date, there have been few cases presented in the literature that have investigated the application of ACT in working with parents and children together in relation to anxiety problems and that have measured the impact on both populations (Hancock et al., 2016). Some researchers have included parents in therapy and reported the impact of parents’ scores on the improvements in their children (Meagher et al., 2017), such as changes in anxiety or emotional and behavioral difficulties. However, they did not report results related to parental value-oriented repertoires, patterns of family accommodation, or improvement in family adjustment to a child’s anxious behavior; as the present study assessed these areas, it provides a significant contribution given the above-stated importance of working on parental practices for improvements in prognoses of child anxiety (Settipani et al., 2013).

It is worth mentioning that this study demonstrates ACT’s versatility for use in both adult and child populations. Following ACT’s laying of foundations of contextual intervention for adults (S. C. Hayes & Strosahl, 2004), a large body of knowledge has shown the usefulness of ACT for working with children and adolescents (E. Harris & Samuel, 2020). The experiential approach to painful private events, or the work around values and identity, typical of ACT (L. W. Coyne et al., 2011; Wilson et al., 2011), are strategies that can teach clients to relate to their own emotions from the perspective of psychological acceptance and to integrate the horizon of personal values in the intervention. In relation to the latter, Miguel attended all the sessions and went along with all their respective dynamics; he was explicit in expressing that he understood and developed actions in his daily life while being committed to his values, despite feeling fear. This is consistent with other studies that have made precise adaptations of ACT clinical tools for use with children and adolescents (see Kennedy et al., 2014).

From a clinical significance perspective, the improvements observed in Miguel’s case highlight important implications for practitioners addressing separation anxiety in children. ACT’s flexibility allows clinicians to tailor interventions to different developmental stages and severity levels, making it applicable across diverse patient populations—from mild to moderate anxiety presentations in school-aged children to more complex cases involving parental accommodation patterns. The concurrent parent–child intervention model demonstrated here may be particularly beneficial for younger children who are more dependent on parental involvement and more susceptible to parental anxiety transmission.

Beyond direct anxiety reduction, several mechanisms likely contributed to the therapeutic gains observed. The development of values-aligned behaviors—as evidenced by Miguel’s willingness to approach feared situations despite discomfort—may have enhanced his sense of meaning and life satisfaction, creating a positive feedback loop that reinforced continued behavioral change. The improvements that can be achieved in parent–child relationship quality, as parents learn to respond with psychological flexibility, likely provided Miguel with a more secure relational foundation for exploring feared situations. Furthermore, the literature has shown that, from a neurobiological perspective, ACT-based exposure to feared situations within a context of acceptance and values-based clarification may promote adaptive neural mechanisms associated with reduced amygdala reactivity and enhanced prefrontal cortex regulation of emotional responses (Hölzel et al., 2011; Kober et al., 2019). These multiple pathways of change suggest that ACT’s benefits extend beyond symptom reduction to encompass broader improvements in psychological well-being and family functioning.

Thus, although there are fewer written works developed with children and adolescents in comparison with work reported with adults (Fang & Ding, 2020), this study adds to the evidence on the effectiveness of ACT in children. This case study is presented primarily as an illustrative clinical example of how ACT processes can be adapted for simultaneous work with parents and children presenting with separation anxiety. Rather than proposing a substantial advance over prior ACT research with youth and families, the contribution of this report lies in offering a detailed description of functional analysis, therapeutic processes, and parallel parent–child interventions that may guide clinicians in adapting ACT tools to similar cases.

The review by Raftery-Helmer et al. (2016) notes that ACT is a treatment model that promises to change problematic interactions between parents and children with anxiety problems. Nevertheless, the authors point out some limitations; they highlight that most studies have focused on outcomes on anxiogenic symptomatology in children, neglecting the importance of examining how intervention in parents changes the studied family interactions. In addition, they stress that it is essential to examine the mechanisms of change that ACT considers to be critical, such as PF and willingness. This case study responds to these limitations since it offers results not only on the depressive and anxiogenic symptomatology in the child, but also on the child’s willingness and committed actions related to their values. In addition, it shows evidence of the impact of the intervention on flexible, values-based parenting according to the assessments made on the mother, based on the functional analysis of the behavior of different members of a family and the interactions among these behaviors. Recently, Flujas-Contreras et al. (2022) also reported on the measures of emotional perspective-taking skills, acceptance, and valued actions in a child, excluding the limitations indicated by Raftery-Helmer et al. (2016). However, it should be noted that the study of Flujas-Contreras et al. (2022) does not introduce a joint mother–child intervention but intervenes only with a mother.

Despite the contributions made here, some limitations are present in this research and should be considered. First, this is a descriptive, single-case report based on pre–post self-report data, without experimental control, repeated assessments, or independent evaluation, which limits the strength and generalizability of the findings. While the intervention duration of 23 sessions falls within the range of recommended ACT protocols for parental interventions, recent meta-analytic evidence suggests that combined ACT interventions with optimal dosage (120 min per session, conducted at least twice weekly, over periods exceeding 8 weeks) may yield enhanced effects for parents of children with anxiety (Guo et al., 2025). Future studies could examine whether comparable benefits might be achieved with shorter or more condensed intervention formats. Additionally, while a follow-up assessment was conducted at 3 months postintervention, the inclusion of a second follow-up assessment at 6 months would have provided a more comprehensive evaluation of long-term maintenance of treatment gains and potential delayed effects, as is common practice in intervention research. Moreover, generalizability is constrained by case-specific characteristics, so the applicability of these findings to other demographic and socioeconomic contexts—such as families with lower socioeconomic status, limited access to mental health services, or different cultural backgrounds with varying conceptualizations of anxiety and parenting—should be examined in future studies. Second, the intervention was carried out with only one of the two parents. The absence of paternal involvement in similar interventions is a frequent limitation suggested in other studies (Flujas-Contreras et al., 2022). In future interventions, it would be necessary to evaluate whether this same intervention protocol reaches similar results in fathers or in families with different gender role configurations. Third, it would have been interesting to assess the impact of the mother’s emotional distress, for example, through standardized anxiety or depression questionnaires, for its possible connection to overcontrolling parental practices that reinforce or maintain the child’s anxious behavior (Raftery-Helmer et al., 2016). Related to this, it would also have been interesting to measure the functioning of the parents as a couple after the intervention, as this had been reported as being deficient. Fourth, self-reports were used without prior training in behavioral observation, so this could have affected the reliability of the results. To avoid this, a pre-intervention session could be carried out with the aim of improving parents’ self-discriminatory competence and/or introducing standardized interaction questionnaires, such as CBCL—Child Behavior Checklist. Last, the treating clinician was also involved in the authorship of the report. Although standardized measures, predefined procedures, and co-author supervision were used to reduce bias, this remains a limitation. Future studies could include independent evaluators, external supervision, or blinded assessments to further minimize potential clinician–author bias.

## 6. Conclusions

In conclusion, this study suggests that interventions for parents and children with anxious topographies under the ACT framework may help to decrease the dysfunctional repertoires of parental accommodation to distress and reduce children’s levels of anxiety and depression, as reported in other studies (Hancock et al., 2016). Moreover, these effects become more significant during the follow-up, similarly to the findings reported by other authors (e.g., Perkins et al., 2023). Strategies derived from contextual–functional science focus specifically on values, which favors medium- to long-term behavior changes, as this is guided by valuable actions rather than avoidance or escape from unpleasant events (L. W. Coyne et al., 2011).

Future research should continue to add evidence on the usefulness of joint parent–child interventions based on ACT to generate value-oriented parental repertoires that improve family adjustment to child anxiety problems. Experimental or multiple baseline studies are recommended where the different components of the intervention can be identified. In this way, the key processes in therapy responsible for generating optimal interventions for the development of flexible parenting can be established.

## Data Availability

Data are contained within the article.
